# Tungsten Oxide Nanorods: An Efficient Nanoplatform for Tumor CT Imaging and Photothermal Therapy

**DOI:** 10.1038/srep03653

**Published:** 2014-01-13

**Authors:** Zhiguo Zhou, Bin Kong, Chao Yu, Xiangyang Shi, Mingwei Wang, Wei Liu, Yanan Sun, Yingjian Zhang, Hong Yang, Shiping Yang

**Affiliations:** 1 The Education Ministry Key Lab of Resource Chemistry and Shanghai Key Laboratory of Rare Earth Functional Materials, Shanghai Normal University, Shanghai 200234, People's Republic of China; 2 College of Chemistry, Chemical Engineering and Biotechnology, Donghua University, Shanghai 210620, People's Republic of China; 3 Department of Nuclear Medicine, Shanghai Cancer Center & Department of Oncology, Shanghai Medical College, Fudan University, Shanghai 200032, People's Republic of China

## Abstract

We report here a facile thermal decomposition approach to creating tungsten oxide nanorods (WO_2.9_ NRs) with a length of 13.1 ± 3.6 nm and a diameter of 4.4 ± 1.5 nm for tumor theranostic applications. The formed WO_2.9_ NRs were modified with methoxypoly(ethylene glycol) (PEG) carboxyl acid *via* ligand exchange to have good water dispersability and biocompatibility. With the high photothermal conversion efficiency irradiated by a 980 nm laser and the better X-ray attenuation property than clinically used computed tomography (CT) contrast agent Iohexol, the formed PEGylated WO_2.9_ NRs are able to inhibit the growth of the model cancer cells *in vitro* and the corresponding tumor model *in vivo*, and enable effective CT imaging of the tumor model *in vivo*. Our “killing two birds with one stone” strategy could be extended for fabricating other nanoplatforms for efficient tumor theranostic applications.

Development of novel nanoparticulate systems for theranostics of cancer that combines both diagnostic and therapeutic functions has increasingly attracted a great deal of attention in recent years^1^,^2^,^3^,^4^,^5^. For effective cancer therapy, it is essential to develop a nanoparticulate system that is able to respond to the local tumor environment or external stimuli such as temperature, pH, magnetic field, light, and enzyme^6^,^7^,^8^,^9^. In particular, light-activated theranostics have been of vital importance. Compared with photodynamic therapy and photo-controlled chemotherapy, photothermal therapy (PTT) makes use of a photosensitizer that converts tissue transparent near-infrared (NIR) light into heat with a minimal attenuation of the energy and prevents undesirable thermal damage to healthy tissue. Over the past decade, a variety of nanoparticulate photothermal agents with a relatively high tissue transparency in the NIR window, such as gold nanostructures (nanorods, nanoshells and nanocages)^10^,^11^,^12^, carbon nanomaterials (carbon nanotubes and nanographenes)^13^,^14^, palladium/germanium nanostructures^15^,^16^,^17^,^18^, copper chalcogenide nanocrystallines^19^,^20^ and other organic nanoparticles^21^,^22^,^23^,^24^ have been intensively investigated. However, in most of the cases, molecular imaging functionalities have not been developed or incorporated within the investigated NP systems for theranostics.

Among the many molecular imaging technologies, X-ray computed tomography (CT) can afford better spatial and density resolution than other imaging modalities. Current iodine-based small molecular CT contrast agents have severe limitations including relatively short circulation times due to rapid renal clearance, renal toxicity, and vascular permeation. Due to the prolonged blood circulating half-life, passive accumulation at the tumor sites *via* enhanced permeation and retention (EPR) effect, and high contrast efficacy, nanoparticulate CT contrast agents comprised of heavy metal elements with a high atomic number have been paid considerable attention recently. A range of NP systems including but not limited to Au^25^,^26^,^27^,^28^,^29^,^30^,^31^, Bi_2_S_3_^32^,^33^, FePt^34^, TaO_x_^35^,^36^ and NaLuF_4_/NaYbF_4_^37^,^38^ nanoparticulate have been developed as effective CT contrast agents. However, most of the developed CT contrast agents have not been simultaneously developed for therapeutic applications.

Generally, theranostic agents are constructed using an integrated platform such as dendrimers^39^,^40^, polymeric micelles^41^, and inorganic nanocarriers^42^,^43^,^44^,^45^,^46^,^47^,^48^, which contain both imaging and therapeutic components (e. g., cancer drug). However, the conventional integration process for theranostic agents requires multiple synthetic steps and additional costs to avoid convoluted behavior and effects *in vivo* and to clear substantial regulatory hurdles. To address these problems, a single component nanomaterial having both imaging and therapeutic functionalities should be an ideal option. Up to now, very few studies have been reported for simultaneous CT imaging and NIR photothermal therapy with a single component agent^49^,^50^.

Due to the high surface-to-volume ratio, large surface energy, and quantum confinement effects, nanostructured tungsten oxide have been developed for electrochromic devices^51^ and gas sensors^52^,^53^, used as a photocatalyst^54^, and applied in field emission displays^55^,^56^. Very recently, plasmonic tungsten oxide NPs have been applied for photothermal therapy^57^. As is well known, tungsten has a higher X-ray absorption coefficient (4.438 cm^2^/kg at 100 keV) than that of iodine (1.94 cm^2^/kg at 100 keV). Therefore, it is reasonable to hypothesize that tungsten oxide NPs may be developed as a theranostic agent for simultaneous tumor CT imaging and photothermal therapy.

In this work, we demonstrated the first use of tungsten oxide nanorods (WO_2.9_ NRs) modified with polyethylene glycol (PEG) for simultaneous CT imaging and NIR photothermal therapy of tumors *in vivo*. The formed PEGylated WO_2.9_ NRs were characterized *via* different techniques. The photothermal therapy performance of the PEGylated WO_2.9_ NRs was confirmed *in vitro* and *in vivo* under irradiation of a 980 nm laser. Further, the *in vivo* tumor CT imaging was confirmed by scanning the mice intratumorally injected with PEGylated WO_2.9_ NRs.

## Results

### Synthesis and characterization of PEGylated WO_2.9_ NRs

**Figure 1:**
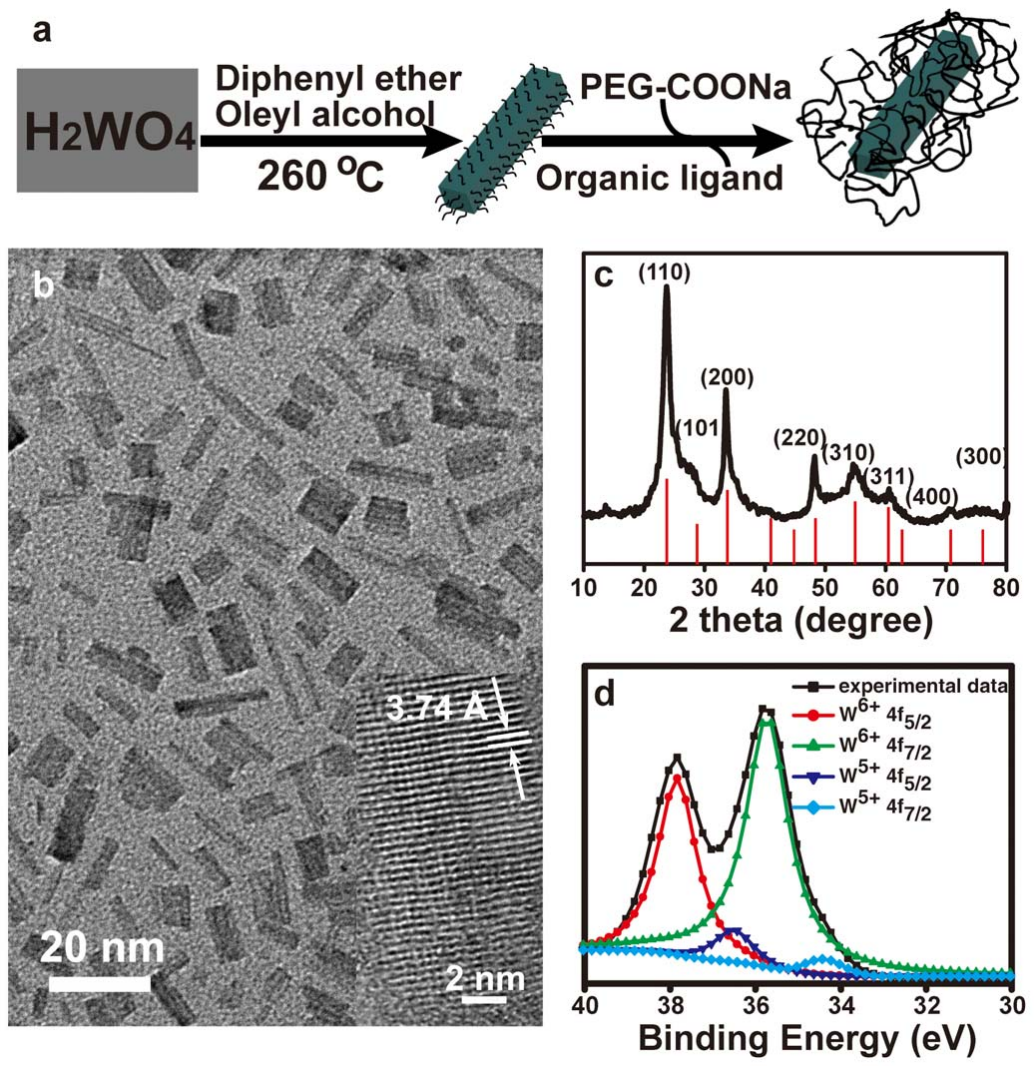
Synthesis and characterization of WO_2.9_ NRs. (a) Schematic illustration of the synthesis of WO_2.9_ NRs and PEGylated WO_2.9_ NRs. (b) TEM image of WO_2.9_ NRs and the corresponding high-resolution TEM image (Inset). (c) Powder X-ray diffraction patterns of WO_2.9_ NRs, as referenced by standard WO_2.9_ phase (JCPDS: 18-1417). (d) W4f core-shell XPS spectra of WO_2.9_ NRs.

WO_2.9_ NRs were synthesized by a modified high-temperature pyrolysis of a cheap and air-stable precursor of tungstic acid in a mixture solvent of oleyl alcohol and diphenyl ether at 260°C under nitrogen atmosphere (Fig. 1a). This process enables the generation of WO_2.9_ NRs that can be well-dispersed in different organic solvents (Fig. S1a). X-ray diffraction (XRD) was used to characterize the crystalline phase of the NRs (Fig. 1c). The peaks shown in the XRD pattern were indexed and the NRs had a nonstoichiometric WO_2.9_ form (JCPDS No.18-1417). A careful examination of a typical transmission electron microscopy (TEM) image (Fig. 1b) show that the formed WO_2.9_ NRs have a length of 13.1 ± 3.6 nm (Fig. S1b) and a diameter of 4.4 ± 1.5 nm (Fig. S1c). A clear lattice fringe with a spacing of ~0.37 nm corresponding to the lattice spacing in the (110) plane of the WO_2.9_ phase can be clearly observed in a typical high-resolution TEM image (inset in Fig. 1b), confirming the crystalline nature of the material. Furthermore, the composition of the NRs was characterized by X-ray photoelectron spectroscopy (XPS). The W4f curve fitting data shown in Fig. 1d reveal that the main peaks at 35.8 and 37.8 eV can be assigned to W^6+^ and the weak peaks located at 36.5 and 34.3 eV be assigned to W^5+^^58^. The existence of W^5+^ may be due to the fact that a part of the W^6+^ is reduced by oleyl alcohol at a high temperature. As a result, a portion of the used oleyl alcohol was oxidized to oleic acid to control the growth of NRs. The presence of oleic acid was confirmed by Fourier transform infrared (FT-IR) spectroscopy (Fig. S1d), where the vibration band at 1655 cm^−1^ assigned to the C = O groups of oleic acid can be clearly observed.

**Figure 2:**
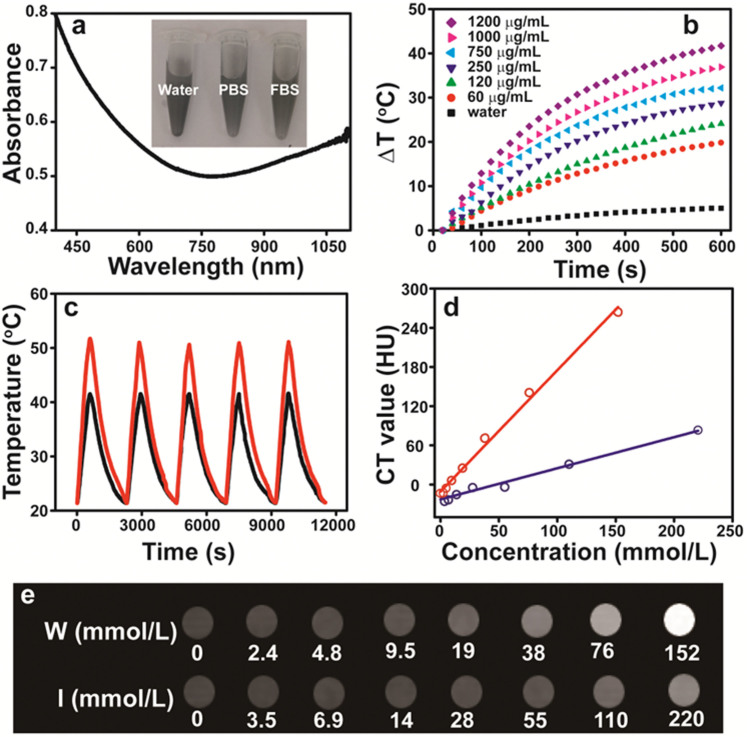
Photothermal and X-ray attenuation property of PEGylated WO_2.9_ NRs. (a) UV-Vis-NIR absorption spectra of PEGylated WO_2.9_ NRs in aqueous solution (100 μg/mL). The inset shows the photographs of WO_2.9_ NRs dispersed in water, PBS, and FBS, respectively. (b) Temperature increase as a function of the concentration of PEGylated WO_2.9_ NRs and irradiation time under the 980 nm laser irradiation (0.25 W/cm^2^). (c) Temperature change of the aqueous solution of PEGylated WO_2.9_ NRs (red: 750 μg/mL, black: 100 μg/mL) over five LASER ON/OFF cycles (0.25 W/cm^2^). (d) CT value (HU) of PEGylated WO_2.9_ NRs and Iohexol as a function of the concentration of tungsten (red) and iodine (blue), respectively. (e) *In vitro* CT images of PEGylated WO_2.9_ NRs (upper panel) and Iohexol (lower panel) with different W or I concentration.

The formed WO_2.9_ NRs stabilized by oleic acid are not dispersible in water, and unable to be used for biomedical applications. Therefore, it is essential to modify their surfaces to endow the NRs with water dispersability and biocompatibility. Through a ligand exchange process, polyoxyethylene chains (PEG) was modified onto the surfaces of the NRs *via* the coordination interaction between carboxylate ions and the tungsten oxide surface. This was confirmed by FT-IR spectrum (Fig. S1d), where the vibration band at 1111 cm^−1^ associated to the C–O–C unit of PEG can be clearly observed. The ligand exchange process was further confirmed by thermal gravimetric analysis (TGA) (Fig. S1e). It is clear that the weight percentage of the organic content changed to ~15% from ~8%, indicating that around 7% PEG has been modified onto the surface of the NRs. Furthermore, we show that the morphology of the NRs after PEGylation does not have any appreciable change in comparison with that of organic ligand-capped NRs (Fig. S1f). Finally, the PEGylated WO_2.9_ NRs were proven to have good dispersability in water, PBS buffer solution, and fetal bovine serum (FBS) and do not precipitate for at least one month (inset in Fig. 2a). The good water solubility and colloidal stability are essential for their further biomedical applications.

### Photothermal effect

The aqueous solution of PEGylated WO_2.9_ NRs (100 μg/mL) showed a blue color with strong absorption in the NIR region (Fig. 2a), which was attributed to the strong localized surface plasmon resonances (LSPR) of the NRs^59^. The strong NIR absorption of PEGylated WO_2.9_ NRs motivated us to investigate their potential application as photothermal agents. The temperature increase of the aqueous solution in the presence of the PEGylated NRs as a function of the NR concentration (60 to 1200 μg/mL) under the 980 nm laser irradiation shows that the solution temperature increase can reach 41.7°C at the NR concentration of 1200 μg/mL, and higher concentration of NRs leads to more prominent temperature increase (Figs. 2b and S2a). In contrast, the water solution in the absence of NRs does not show any obvious temperature increase under similar experimental conditions (Fig. 2b). To assess the NIR photostability of PEGylated WO_2.9_ NRs, five cycles of Laser on/off were performed by irradiating the aqueous solution of PEGylated WO_2.9_ NRs *via* a 980 nm laser for 10 min (Laser on), followed by cooling down to room temperature without NIR laser irradiation for 30 min (Laser off). As shown in Fig. 2c, the temperature increases of 20.1°C and 30.3°C were able to be achieved after the first laser on for the NR concentration of 100 and 750 μg/mL, respectively. No significant change in the temperature increase was observed after five cycles. Furthermore, the absorbance of the NRs (180 μg/mL) at 980 nm remained stable even after ten cycles of laser irradiation (Fig. S2b).

### X-ray attenuation property

Considering the high atomic number and X-ray absorption coefficient of tungsten, the contrast efficacy of PEGylated WO_2.9_ NRs as a CT contrast agent was evaluated (Fig. 2d). Iohexol, a conventional iodine-based CT contrast agent was used as control. The CT value, measured in Hounsfield units (HU), increased linearly as a function of the concentration of W or I (Fig. 2d). However, the increasing trend of the NRs (the slope of the CT value for PEGylated WO_2.9_ NRs was ~1.9) was much higher than that of Iohexol with a slope of CT value of ~0.5. At equal concentrations of W or I element, the CT contrast enhancement of PEGylated WO_2.9_ NRs was much higher than that of Iohexol, which is primarily due to the higher X-ray attenuation coefficient of W than that of I.

### Photothermal therapy of cancer cells *in vitro*

The high photothermal conversion efficiency of PEGylated WO_2.9_ NRs prompted us to evaluate the feasibility to use them as a photothermal agent for cancer therapy. Firstly, HeLa cells (a a human epithelial cervical cancer cell line) and L929 cells (a normal mouse fibroblast cell line) were used to evaluate the cytotoxicity of the PEGylated WO_2.9_ NRs *via* a standard 3-(4,5-dimethylthiazol-2-yl)-2,5-diphenyltetrazolium bromide (MTT) cell viability assay (Fig. S4a). It is clear that the viability of both HeLa and L929 cells treated with the NRs is all greater than 80% even at the NR concentration of 500 μg/mL. This suggests that the PEGylated WO_2.9_ NRs are quite cytocompatible at the NR concentration up to 500 μg/mL. It is essential to check the hemocompatibility of the material, especially under that the circumstance that requires the material to contact blood. We show that at the NR concentration of 400 μg/mL, the hemolysis percentage of PEGylated WO_2.9_ NRs was only 2.6%, much less than the threshold value of 5% (Figs. S4b–S4d), suggesting their excellent hemocompatibility. These studies demonstrate that in the given concentration range, the PEGylated WO_2.9_ NRs are cytocompatible and hemocompatible, which is essential for their further biomedical applications.

**Figure 3:**
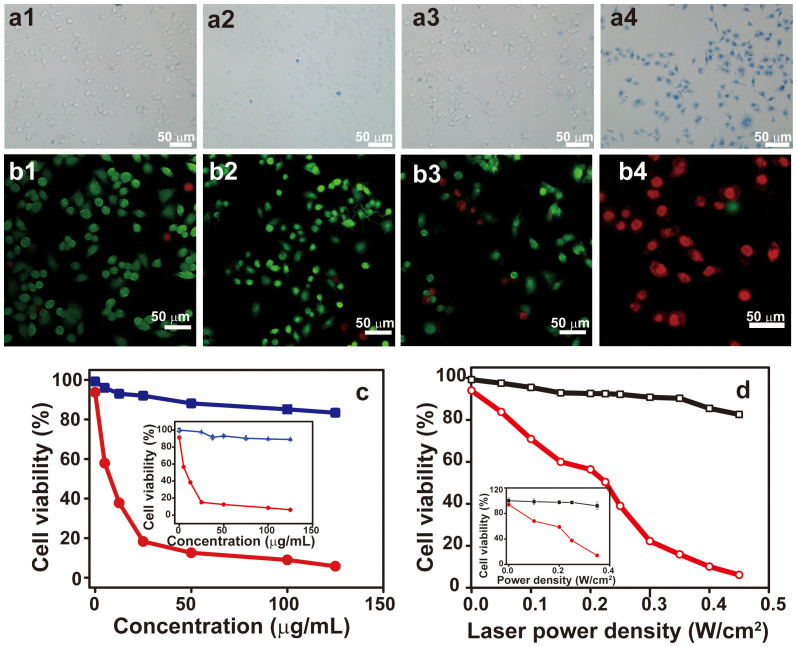
Photothermal ablation of HeLa cells *in vitro* using PEGylated WO_2.9_ NRs under 980 nm laser irradiation. Optical images stained by trypan blue (a) and confocal fluorescence images stained by Calcein AM/PI (b). (a1) and (b1), control HeLa cells without treatment. (a2) and (b2), HeLa cells treated with the 980 nm laser irradiation for 8 min (0.35 W/cm^2^) in the absence of PEGylated WO_2.9_ NRs. (a3) and (b3), HeLa cells incubated with PEGylated WO_2.9_ NRs (50 μg/mL) without laser irradiation. (a4) and (b4), HeLa cells incubated with PEGylated WO_2.9_ NRs (50 μg/mL) under the 980 nm laser irradiation for 8 min (0.35 W/cm^2^). (c) Cell viability determined by FACS and MTT assay (inset) under different concentrations of PEGylated WO_2.9_ NRs with (red) or without (blue) the 980 nm laser irradiation for 8 min (0.35 W/cm^2^). (d) Cell viability determined by FACS and MTT assay (inset): cells were treated with 980 nm laser irradiation at different power density for 8 min with (red) or without (black) PEGylated WO_2.9_ NRs (50 μg/mL).

We next evaluated the capability of using PEGylated WO_2.9_ NRs to photothermally ablate cancer cells by typan blue staining under laser irradiation. As shown in the optical microscopic images in Fig. 3a, there were no obvious changes in the control groups of HeLa cells with or without laser irradiation (Figs. 3a1 and 3a2). Moreover, negligible cell ablation was observed for cells incubated with PEGylated WO_2.9_ NRs without laser irradiation (Fig. 3a3). In contrast, the HeLa cells incubated with PEGylated WO_2.9_ NRs (50 μg/mL) were stained blue after laser irradiation for 8 min (Fig. 3a4), indicating that HeLa cells were ablated by NIR laser irradiation.

The photothermal ablation of cancer cells was also confirmed by laser confocal fluorescence microscopy. After laser irradiation, HeLa cells were co-stained with Calcine AM and propidium iodide (PI) to differentiate live (green) and dead cells (red), respectively. The majority of HeLa cells were able to be ablated after treated with 50 μg/mL PEGylated WO_2.9_ NRs under laser irradiation at 0.35 W/cm^2^ for 8 min, suggesting that the PEGylated WO_2.9_ NRs are able to kill HeLa cells *via* photothermal destruction (Fig. 3b4). In contrast, the HeLa cells without treatment were not affected by laser irradiation under similar experimental conditions (Fig. 3b3). These results corroborate with the above optical microscopic imaging data.

We further quantitatively investigated the photothermal ablation of HeLa cells by MTT assay and flow cytometry (FACS). HeLa cells incubated with 5 to 125 μg/mL PEGylated WO_2.9_ NRs for 4 h in 12-well plates were irradiated under a 980 nm laser at 0.35 W/cm^2^ for 8 min. Then, the cell viability was measured. As controls, HeLa cells were treated similarly with PEGylated WO_2.9_ NRs without laser irradiation and were laser irradiated in the absence of PEGylated WO_2.9_ NRs. As shown in Fig. 3c, the cell viability of the two control groups was higher than 85%, indicating that PEGylated WO_2.9_ NRs or the 980 nm laser irradiation alone have negligible effect on HeLa cells. However, when HeLa cells were incubated with 5–25 μg/mL PEGylated WO_2.9_ NRs and irradiated with a 980 nm laser at 0.35 W/cm^2^, the cell viability decreased rapidly with the NR concentration. At the NR concentration of 25 μg/mL, the cell viability decreased to 15.0% ± 1.4% (measured by MTT assay), which is comparable to ~18.3% analyzed by FACS. The cell viability decreased very slowly at higher concentrations. From 25 to 125 μg/mL, the cell viability decreased from 15.0% ± 1.4% to 6.2% ± 0.6% as measured by MTT assay, which can be confirmed to be from ~18.3% to ~5.8% as analyzed by FACS. The median lethal dose induced by a 980 nm laser at 0.35 W/cm^2^ was ~5 μg/mL. Furthermore, photothermal cytotoxicity was enhanced by increasing the NIR laser power density. At an NR concentration of 50 μg/mL, the viability of HeLa cells decreased from 68.1% ± 0.8% to 13.0% ± 0.2% as measured by MTT assay (from ~70.9% to ~6.1% as analyzed by FACS) with an increase in the laser power density from 0.1 to 0.45 W/cm^2^. The median lethal power density for HeLa cells incubated with 50 μg/mL PEGylated WO_2.9_ NRs was ~0.20 W/cm^2^. These experimental findings demonstrate that the combination of PEGylated WO_2.9_ NRs and NIR laser irradiation is able to ablate cancer cells *in vitro* in a localized manner. Therefore, PEGylated WO_2.9_ NRs have a great potential to be used for *in vivo* photothermal tumor therapy.

**Figure 4:**
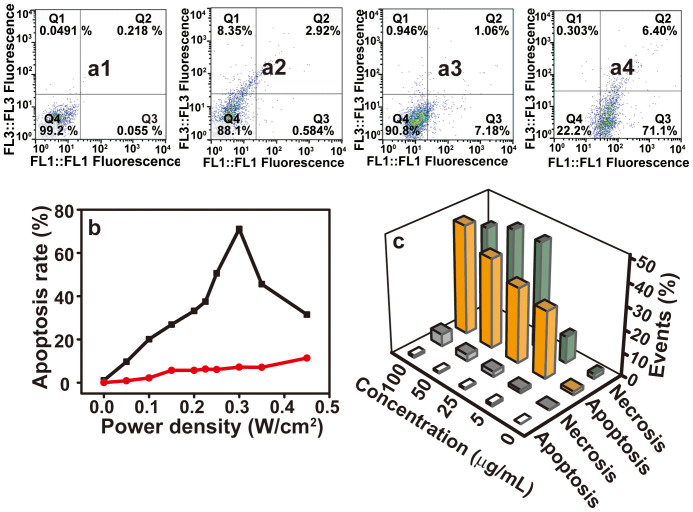
The mechanism of photothermal ablation of HeLa cells using PEGylated WO_2.9_ NRs determined by FACS with 980 nm laser irradiation. Representative FACS plots. (a1), control HeLa cells without treatment. (a2), HeLa cells incubated with 50 μg/mL PEGylated WO_2.9_ NPs without laser irradiation. (a3), HeLa cells after 980 nm laser irradiation for 8 min (0.30 W/cm^2^) in the absence of PEGylated WO_2.9_ NRs. (a4), HeLa cells incubated with 50 μg/mL PEGylated WO_2.9_ NPs after 980 nm laser irradiation for 8 min (0.30 W/cm^2^). (b) apoptosis rate of HeLa cells incubated with (black) or without (red) PEGylated WO_2.9_ NPs (50 μg/mL) at different power density of 980 nm laser irradiation for 8 min. (c) apoptosis and necrosis rate of HeLa cells incubated with different concentrations of PEGylated WO_2.9_ NPs with (orange and green bars) or without (white and grey bars) 980 nm laser irradiation for 8 min (0.35 W/cm^2^).

To clarify the cell death mode after photothermal treatment, an annexin V-FITC (AV))/PI assay was conducted by FACS. The quantity of apoptotic cells was determined by the percentage of AV+/PI−, while the quantity of necrotic cells was determined by the percentages of AV+/PI−. As shown in Figs. 4a1–4a4, the apoptosis rate of HeLa cells is ~0.6% when incubated with PEGylated WO_2.9_ NRs at a concentration of 50 μg/mL and ~7.2% after laser irradiation at 0.30 W/cm^2^. Therefore, neither PEGylated WO_2.9_ NPs nor 980 nm laser irradiation alone exerted obvious destructive effects to HeLa cells. In sharp contrast, the apoptosis rate of cells incubated with PEGylated WO_2.9_ NPs under the above conditions under laser irradiation was ~71% (Fig. 4), while the necrosis rate was only ~6.4%. Therefore, under this condition, the mechanism of *in vitro* photothermal therapy is mainly triggered by cell apoptosis, not necrosis. The power density- and concentration-dependence of the cell death mode of HeLa cells were further investigated in detail, as shown in Figs. 4b and 4c. As a control, the apoptosis rate of HeLa cells treated only with PEGylatedWO_2.9_ NRs at the highest NR concentration of 100 μg/mL for 4 h is only ~1.0%. On the other hand, in the absence of PEGylated WO_2.9_ NRs, the apoptosis rate was ~11.4% when irradiated with the highest power density of 0.45 W/cm^2^ for 8 min. The control experiments demonstrate that the PEGylated WO_2.9_ NRs or 980 nm laser irradiation alone do not induce any obvious cell apoptosis. In the presence of PEGylated WO_2.9_ NRs with a high concentration (50 μg/mL), the apoptosis rate of HeLa cells increased from ~9.7% to ~71% with an increase in the laser power density from 0.05 to 0.30 W/cm^2^, respectively. However, there was no obvious increase in the percentage of necrotic cells. To our surprise, with the increase in the laser power density from 0.30 to 0.45 W/cm^2^, the apoptosis rate decreased from ~71% to 31.5%, with a significant increase in the necrosis rate from 6.4% to 58.9%. These experimental findings demonstrate for the first time that photothermal therapy occurs *via* an apoptosis-mediated mechanism with a low laser power density; however, with a high laser power density, apoptosis and necrosis modes were simultaneously observed. The apoptosis and necrosis modes with a laser power density of 0.35 W/cm^2^ were further confirmed by increasing the concentration of PEGylated WO_2.9_ NRs (Fig. 4c). For instance, the apoptosis and necrosis rates were 29.3% and 11.7% in the presence of PEGylated NRs (5 μg/mL), respectively, while the apoptosis and necrosis rates increased to 47.5% and 41.1% in the presence of 100 μg/mL PEGylated NRs, respectively.

### CT imaging and photothermal therapy of a tumor model *in vivo*

**Figure 5:**
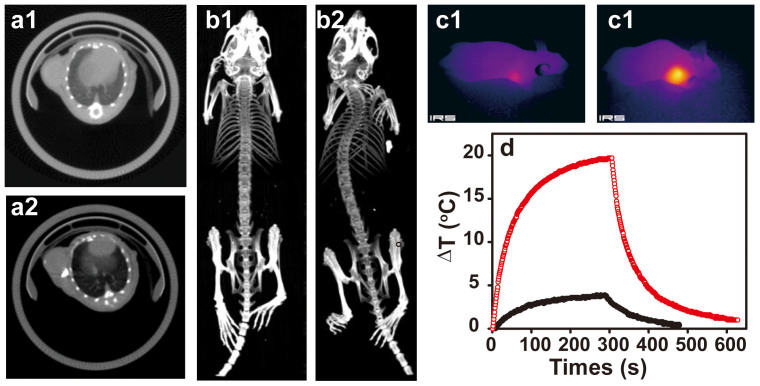
CT and photothermal imaging of a tumor model *in vivo*. (a) *In vivo* X-ray transverse CT images of tumor (a1and a2) and 3-D renderings of *in vivo* CT images (b1 and b2) before (a1 and b1) and after (a2 and b2) intratumoral injection of PEGylated WO_2.9_ NRs (20 mg/kg). (c) *In vivo* photothermal images of HeLa tumor-bearing mice without (c1) and with (c2) PEGylated WO_2.9_ NRs (20 mg/kg) exposed a 980 nm laser for 5 min. (d) temperature change of HeLa tumor-bearing mice without (black)and with (red) PEGylated WO_2.9_ NRs (20 mg/kg) exposed a 980 nm laser for 10 min.

As a proof-of-concept, we tested the feasibility to use PEGylated WO_2.9_ NPs as a CT contrast agent for *in vivo* tumor imaging. PEGylated WO_2.9_ NRs dispersed in physiological saline were intratumorally injected into the xenografted HeLa tumor model in a nude mouse with a dosage of 20 mg/kg of body weight. The tumor signal was clearly enhanced immediately after injection (Figs. 5a1, 5a2, Figs. 5b1 and 5b2). The CT value determined from a 0.4 mm^3^ region of the tumor was measured to be 235 ± 30 HU before injection. The injection of PEGylated WO_2.9_ NPs gave rise to a significant tumor CT value increase (6996 ± 1735 HU), indicating that the developed PEGylated WO_2.9_ NPs are able to be used as a contrast agent for tumor CT imaging.

Motivated by the obvious photothermal property of PEGylated WO_2.9_ NRs *in vitro* under the 980 nm laser irradiation, we then investigated the potential to use them to photothermally ablate HeLa tumor model *in vivo*. Tumor bearing mice were intratumorally injected with PEGylated WO_2.9_ NRs (20 mg/kg) and then irradiated with a 980 nm laser. The local temperature change was monitored by an infrared thermal camera, and plotted as a function of the irradiation time (Fig. 5d). The surface temperature of the tumor injected with PEGylated WO_2.9_ NRs rapidly increased by 20°C under 980 nm laser irradiation at 0.35 W/cm^2^ for 5 min, which was high enough to kill the tumor *in vivo*. The temperature of the tumor region without injection of PEGylated WO_2.9_ NRs was not obviously affected. Therefore, besides CT imaging, PEGylated WO_2.9_ NRs can be used as a good photothermal imaging agent for tumor imaging *in vivo*.

**Figure 6:**
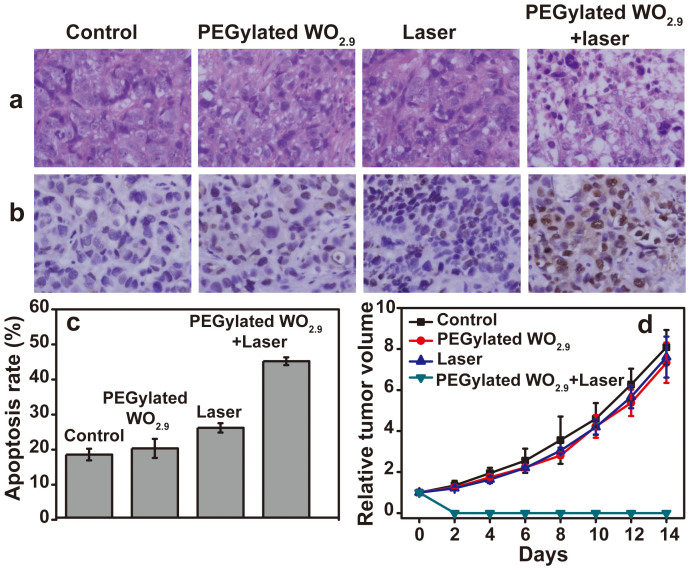
Photothermal Therapy *in Vivo*. (a) H&E-staining images of representative specimens at ×100 magnification. (b) TUNEL staining images of representative specimens at ×100 magnification. (c) TUNEL positive percentage of tumor tissue measured by TUNEL assay. (d) The relative tumor volume as a function of time.

To further confirm the tumor ablation resulted from the photothermal effect of PEGylated WO_2.9_ NRs, the damage levels of tumor tissues were examined using hematoxylin and eosin (H&E) staining after different treatments. The tumor tissues treated with either PEGylated WO_2.9_ NRs or laser irradiation alone showed a similar damage level to the untreated tumor. As expected, a high damage level was observed in the tumor tissue treated with a 980 nm laser and PEGylated WO_2.9_ NRs. The photothermal therapy for tumor tissue was further quantitatively confirmed by TUNEL staining after various treatments. As shown in Fig. 6, 20% ± 2.7% and 26% ± 2.7% TUNEL positive cells were found after treatment with PEGylated WO_2.9_ NRs without laser irradiation and after treatment with the laser irradiation in the absence of PEGylated WO_2.9_ NRs, respectively. However, after laser irradiation, the quantity of TUNEL positive cells increased to 45% ± 1.1% after treated with the NRs.

The photothermal therapeutic efficacy of PEGylated WO_2.9_ NRs by a 980 nm laser was further evaluated. Four groups of HeLa tumor-bearing mice with 5 mice per group were used in our experiment. All mice were alive during the period of the photothermal therapy. The relative tumor volumes (V/V_0_) were calculated for each mouse and plotted as a function of time (Fig. 6d). The relative tumor volumes for the group of intratumorally injected with PEGylated WO_2.9_ NRs only and the group exposed to the NIR laser irradiation alone were similar to that of the group injected with saline, suggesting that injection of PEGylated WO_2.9_ NRs only or irradiation of the 980 nm laser alone is unable to inhibit the tumor growth. As expected, tumor growth in the group injected with PEGylated WO_2.9_ NPs and exposed the NIR laser irradiation were completely inhibited (Fig. S10). These results further demonstrate that the combination of PEGylated WO_2.9_ NRs and NIR irradiation is essential for effective photothermal therapy of tumors.

## Discussion

Generally, non-stoichiometric tungsten oxide nanomaterials are synthesized from the precursor of tungsten ethoxide and tungsten chloride, which are of high cost and very sensitive to the moisture^57^,^59^. In our procedure, the cheap and stable tungsten acid was adopted, which was convenient to handle under the conventional experimental conditions. The decomposable inorganic acid precursor may be developed as a general precursor for the synthesis of other nanomaterials. The developed PEGylated WO_2.9_ NRs were quite photostable under the NIR laser irradiation when compared with the classical photothermal agents (e.g., Au NRs). Our results reveal that five cycles of laser on/off (0.25 W/cm^2^) do not lead to any significant changes of the temperature and the absorbance at 980 nm for PEGylated WO_2.9_ NRs; however, the same process of laser on/off results in vanished absorption peak of Au NRs in the NIR region and a gradual decrease of temperature (Fig. S3). Furthermore, the contrast efficiency of PEGylated WO_2.9_ NRs was ~ 4-fold higher than that of the clinical CT contrast agent (Iohexol). As a result, to obtain an equivalent contrast enhancement, PEGylated WO_2.9_ NRs can be used at a much lower dose, which can avoid the adverse side effects of higher dose administration when compared with the clinically used iodinated contrast agent. It is interesting to note that in our study, we selected HeLa cells (a human epithelial cervical cancer cell line) for theranostics because HeLa cells have been widely used as model cancer cell line. The success of our work may be beneficial for theranostics of cervical cancer, as well as other types of cancer. Due to the significant nonspecific cellular uptake of the PEGylated WO_2.9_ NRs possibly *via* two dictinct mechanisms (phagocytosis and diffusion via cell walls)^29^, the cancer cells are able to be significantly inhibited after photothermal treatment. For effective tumor CT imaging and photothermal ablation, we selected to use intratumoral injection of the PEGylated WO_2.9_ NRs. This is because the developed PEGylated WO_2.9_ NRs do not possess active targeting ligands and the passive targeting of the particles via enhanced permeability and retention (EPR) effect after intravenous injection of the particles was proven to be not sufficiently effective by our CT imaging experiment (Fig. S5). For non-targeted NPs or targeted NPs with a size more than 100 nm, intratumoral injection may be selected as an effective administration route for tumor imaging^31^ and therapy^60^.

Though many types of nanoparticulate theranostic agents that are integrated with both imaging agents and therapeutic components have been reported^39^,^40^,^41^,^42^,^43^,^44^,^45^,^46^,^47^,^48^, the developed one-component theranostic agents similar to the one developed in our study are quite limited. For example, metal ferrite nanoparticles for both MR imaging and magnetic hyperthermia^61^,^62^, Fe_3_O_4_ nanoparticles for both MR imaging and NIR photothermal therapy^63^ and and porphysomes for photoacoustic imaging and photodynamic therapy^64^ have been developed. Compared to the conventional integration procedure, our ‘killing two birds with one stone' strategy is quite convenient and economic, and may be used as a new theranostic agents for different biomedical applications.

In summary, we demonstrated the use of PEGylated NRs for simultaneous X-ray CT imaging and NIR photothermal therapy of tumors with a "killing two birds with one stone" strategy. Combining the advantage of deep tissue spatial penetration without tissue damage for CT imaging, and that of minimal attenuation of the energy and undesirable heating of healthy tissue for PTT, the higher X-ray attenuation coefficient of PEGylated WO_2.9_ NRs than that of iodine-based small molecular CT contrast agent enables them to be used for sensitive CT imaging of tumors. On the other hand, with the high intensity absorption in the NIR region, the developed PEGylated WO_2.9_ NRs are able to have high photothermal conversion, amenable for photothermal ablation of cancer cells *in vitro* and *in vivo*. The developed PEGylated WO_2.9_ NRs may be further functionalized with different targeting ligands for targeted CT imaging-guided phototheraml therapy of different types of cancer.

## Methods

### Materials

Oleyl alcohol was purchased from TCI, diphenyl ether was purchased from Sigma-Aldrich. Tungstic acid was purchased from Sinopharm Chemical Reagent Co., Ltd. Methoxypoly(ethylene glycol) carboxyl acid (2000) was purchased from Shanghai Seebio Biotech. All reagents were used without further purification. Water used in all experiments was purified using a Milli-Q Plus 185 water purification system (Millipore, Bedford, MA) with resistivity higher than 18 MΩ cm.

### Characterization

X-ray diffraction (XRD) was performed using a Rigaku DMAX 2000 diffractometer equipped with Cu/Kα radiation at a scanning rate of 4°/min in the 2θ range of 10 to 80° (λ = 0.15405 nm, 40 kV, 40 mA). TEM was carried out using a JEOL JEM-2010 transmission electron microscope operating at 200 kV. TEM samples were prepared by depositing a diluted NR suspension (1000 μg/mL, 5 μL) onto a carbon-coated copper grid and air-dried before the measurements. FT-IR spectra were collected on a Nicolet Avatar 370. The samples were pelletized with KBr before measurements. UV-Vis-NIR absorption spectra were recorded on a DU 730 UV-visible spectrophotometer (Nucleic acid, protein analyzer) at room temperature. PEGylated WO_2.9_ NRs were dispersed in water before the experiments. The tungsten concentration was determined by inductively coupled plasma atomic emission spectroscopy (ICP-AES) (VISTAMPXICP VARIAN, AMERICA). X-ray photoelectron spectroscopy (XPS) was performed on a Kratos AXIS-165 surface analysis system. Thermal gravimetric analysis (TGA) was carried out on a Perkin-Elmer TGA-2 thermogravimetric analyzer under nitrogen from 100°C to 800°C at 10°C min^−1^.

### Synthesis of WO_2.9_ NRs

In a typical process, tungstic acid (0.75 mmol, 187.5 mg) was dissolved in a mixture solvent of oleyl alcohol (20 mL) and diphenyl ether (30 mL) in a 100 mL three-neck flask. The solution was degassed under a nitrogen flow, heated to 260°C at 10 K/min under vigorous magnetic stirring, and kept for 60 min at this temperature before it was cooled down to room temperature. After centrifugation at 10000 rpm for 6 min, the supernatant was removed and a blue precipitate was obtained. The resulting blue precipitate was washed with ethanol for three times to acquire pure WO_2.9_ NRs. Finally, the obtained NRs were stored in 40 mL chloroform before use.

### Preparation of PEGylated WO_2.9_ NRs

In a typical process, methoxypoly(ethylene glycol) carboxyl acid (56 mg) was reacted with NaOH (1 mg) in 20 mL of chloroform for 4 h, followed by addition of a chloroform solution of WO_2.9_ NRs (10 mL, 5000 μg/mL). The solution mixture was stirred overnight. The formed PEGylated WO_2.9_ NRs were purified by washing with ethanol and water each for three times before further characterization.

### Photothermal experiments of PEGylated WO_2.9_ NRs

An aqueous suspension (2 mL) containing PEGylated WO_2.9_ NRs with different concentrations was put in a quartz cuvette with an optical path length of 0.5 cm. The cuvette was illuminated by a 980 nm laser (Shanghai Xilong Optoelectronics Technology Co., Ltd.) with a power density of 0.25 W/cm^2^ for 600 seconds. The increase in temperature was monitored by a digital thermocouple device.

### Cell culture

A human cervical carcinoma cell line (HeLa cells) was provided by Shanghai Institute of Biological Sciences, the Chinese Academy of Sciences. HeLa cells were cultured in RPMI-1640 medium (Thermo, USA) supplemented with 10% FBS (Gibco, USA) and 1% penicillin-streptomycin (Thermo, USA) at 37°C and 5% CO_2_. Cells were generally plated in cell culture flask (Corning, USA) and allowed to adhere for 24 h, then harvested by treatment with 0.25% trypsin-EDTA solution (Gibco, USA).

### MTT assay

*In vitro* cytotoxicity of PEGylated WO_2.9_ NRs was evaluated by methyl thiazolyl tetrazolium (MTT) viability assay of HeLa cells. Cells were seeded into a 96-well cell culture plate at a density of 5 × 10^4^ cells/well in RPMI-1640 medium supplemented with 10% FBS and 1% penicillin-streptomycin at 37°C and 5% CO_2_ for 24 h. The next day, the cells were incubated with PEGylated WO_2.9_ NRs with different concentrations (0, 25, 50, 75, 100, 150, 200, 250, 500, and 1000 μg/mL in RPMI-1640) for 12 h or 24 h at 37°C under 5% CO_2_. Thereafter, MTT (20 μL, 5000 μg/mL) was added to each well and the plate was incubated for additional 4 h at 37°C. After removal of the medium, the purple formazan product was dissolved with DMSO for 15 min. Finally, the optical absorption of formazan at 490 nm was measured by an enzyme-linked immunosorbent assay reader (Multiskan MK3, USA.), and the background subtraction at 690 nm was applied.

### Hemolysis assay

Fresh human blood stabilized with heparin was provided by Shanghai blood center. The healthy red blood cells (HRBCs) were isolated from fresh human blood by centrifugation at 2000 rpm for 10 min and purified by five successive rinsing steps with PBS. After that, the suspension of HRBCs was diluted 10 times with PBS. The diluted HRBC suspension (0.3 mL) was added to 1.2 mL of water (positive control), PBS (negative control), and PBS containing PEGylated WO_2.9_ NRs with a concentration ranging from 50 to 400 μg/mL, respectively. After a gentle shaking, the mixtures were kept for 2 h at room temperature. After centrifugation of the mixtures for 1 min, the absorbance of the supernatants was recorded by a UV-Vis spectrophotometer. The hemolysis percentages of the samples were calculated by dividing the difference in absorbances at 541 nm between the samples and the negative control by the difference in absorbances at 541 nm between the positive and negative controls.

### X-ray attenuation measurements

PEGylated WO_2.9_ NRs or the clinically used CT contrast agent Iohexol with different concentrations were prepared in 1.5-mL Eppendorf tubes and placed in a self-designed scanning holder. CT scans were performed using a GE Light Speed VCT 64-detector CT (GE Amersham Healthcare System, Milwaukee, WI). Imaging parameters were as follows: slice thickness, 0.625 mm; pitch, 0.984:1; voltage, 80 kV; current, 500 μA; field of view, 512 × 512; gantry rotation time, 0.4 s; table speed, 40 mm/rotation; view, 84 × 84.

### Photothermal ablation of HeLa cells *in vitro*

To investigate the photothermal ablation of HeLa cells by MTT assay under a 980 nm laser with different power density, 500 μL PBS or a solution of PEGylated WO_2.9_ NRs (100 μg/mL in PBS buffer solution) were added to a 12-well cell culture plate containing HeLa cells with a density of 5 × 10^5^ cells/well in RPMI-1640 medium (500 μL) supplemented with 10% FBS and 1% penicillin-streptomycin. Then, the HeLa cells were incubated for 4 h at 37°C and 5% CO_2_. The adherent cell solution was exposed to a 980 nm laser for 8 min with different laser power density (0, 0.10, 0.20, 0.25, 0.35 and 0.45 W/cm^2^, respectively). After the laser irradiation, HeLa cells were cultured for additional 1 h for MTT assay. Similarly, to investigated the photothermal ablation of HeLa cells as a function of the concentration of the PEGylated WO_2.9_ NRs by MTT assay, PBS (500 μL) or PEGylated WO_2.9_ NRs with different concentrations (0, 10, 25, 50, 100, 200 and 250 μg/mL, respectively) were added, then the HeLa cells were incubated for 4 h at 37°C and 5% CO_2_. The adherent cell solution was exposed to a 980 nm laser for 8 min with a power density of 0.35 W/cm^2^. After the laser irradiation, HeLa cells were cultured for additional 1 h for MTT assay. All measurements were done in triplicate.

### Typan blue staining

PBS (100 μL) or PEGylated WO_2.9_ NR solution (100 μg/mL in PBS buffer solution) were added to a 96-well cell culture plate containing HeLa cells with a density of 5 × 10^4^ cells/well in RPMI-1640 medium (100 μL) supplemented with 10% FBS and 1% penicillin-streptomycin, then the HeLa cells were incubated for 4 h at 37°C and 5% CO_2_. The adherent cell solution was exposed to a 980 nm laser for 8 min with a power density of 0.35 W/cm^2^, then the HeLa cells were cultured for additional 1 h. After that, HeLa cells were stained with 0.4% trypan blue solution for 3 min. After removal of the medium, the adherent cells were washed with PBS for three times. Cell morphology of the adherent cells in PBS (100 μL) was observed by an inverted optical microscope (Olympus, IX71, Japan) with a magnification of 200×. Cells stained by trypan blue were counted as dead cells. Each experiment was carried out in triplicate.

### Laser scanning confocal microscopy

Confocal microscopic imaging was performed with a Leica TCS SP5 inverted microscope (DMI 6000B, Solms, Germany). A 63× oil-immersion objective lens was used. For calcein-AM/PI assay, 500 μL PBS or PEGylated WO_2.9_ NRs solution (100 μg/mL in PBS buffer solution) were added to a 12-well cell culture plate containing HeLa cells with a density of 5 × 10^5^ cells/well in RPMI-1640 medium (500 μL) supplemented with 10% FBS and 1% penicillin-streptomycin, then the HeLa cells were incubated for 4 h at 37°C and 5% CO_2_. The adherent cell solution was exposed to a 980 nm laser for 8 min with a power density of 0.35 W/cm^2^, then the HeLa cells were cultured for additional 1 h. After that, a mixture solution (0.8 mL) containing calcein-AM (2 μmol/L) and PI (4 μmol/L) was added to the cells, then the cells were incubated for additional 15 min. Calcein-AM and PI was excited by the 488 nm and 543 nm lasers, respectively.

### Flow cytometry

To investigate the photothermal ablation of HeLa cells irradiated with the 980 nm laser with different power intensities by FACS, PBS (500 μL) or PEGylated WO_2.9_ NR solution (100 μg/mL in PBS buffer solution) were added to a 12-well cell culture plate containing HeLa cells with a density of 5 × 10^5^ cells/well in RPMI-1640 medium (500 μL) supplemented with 10% FBS and 1% penicillin-streptomycin, then the HeLa cells were incubated for 4 h at 37°C and 5% CO_2_. The adherent cell solution was exposed to a 980 nm laser for 8 min with different laser power density (0, 0.10, 0.20, 0.25, 0.35 and 0.45 W/cm^2^, respectively). After the laser irradiation, HeLa cells were cultured for additional 1 h, washed twice with PBS, and harvested by treatment with 0.25% trypsin-EDTA solution. After centrifugation, the obtained HeLa cells were suspended in PBS solution and analyzed with a flow cytometer (Beckman Coulter, Quanta SC, USA). The collected data were analyzed using Flow Jo software 7.6.5. Similarly, to investigate the photothermal ablation of HeLa cells as a function of the concentration of the PEGylated WO_2.9_ NRs by FACS, PBS (500 μL) or PEGylated WO_2.9_ NR solution with different concentrations (0, 10, 25, 50, 100, 200 and 250 μg/mL in PBS solution, respectively) were added. The adherent cell solutions were exposed to a 980 nm laser for 8 min with a power density of 0.35 W/cm^2^. After the laser irradiation, HeLa cells were cultured for additional 1 h, washed twice with PBS, and harvested by treatment with 0.25% trypsin-EDTA solution. After centrifugation, the obtained HeLa cells were suspended in PBS solution (1 mL) and analyzed with a flow cytometer (Beckman Coulter, Quanta SC, USA). The collected data were analyzed by using Flow Jo software 7.6.5.

### Animal experiments

HeLa tumor-bearing nude mice (~20 g body weight) were purchased from Shanghai SLAC Laboratory Animal Co., Ltd. All the animal experiments were approved by the Institutional Animal Care and Use Committee of Shanghai Normal University and carried out ethically and humanely. The nude mice were used for further experiments when the tumor volume reached about 360 mm^3^.

### In vivo CT imaging

Hela tumor-bearing nude mice were first anesthetized by intraperitoneal injection of chloral hydrate solution (10 wt%), then PEGylated WO_2.9_ NRs (200 μL, 20 mg/kg body weight) or physiological saline (200 μL) were intratumorally injected into the xenografted HeLa tumor model in a nude mouse with a 0.5-gauge needle. The needle was maintained in the tumor for ten seconds to allow the tumor to accommodate the additional fluid. After intratumoral injection, CT imaging was obtained on a Siemens Biograph mCT scanner. Imaging parameters were as follows: voltage, 60 kV; current, 500 μA; field of view, 512 × 512; gantry rotation time, 0.5 s.

### Photothermal imaging *in vivo*

Nude mice bearing HeLa tumors were first anesthetized by intraperitoneal injection of chloral hydrate solution (10 wt%), then 200 μL PEGylated WO_2.9_ NRs (20 mg/kg) or physiological saline was intratumorally injected into the xenografted HeLa tumor model in a nude mouse. After 1 h, the tumor in the mice was exposed to a 980 nm laser with a power density of 0.35 W/cm^2^ for 5 min for photothermal imaging on FLIR A300 (USA).

### H&E and tunel staining

Nude mice bearing HeLa tumors were intratumorally injected into the xenografted HeLa tumor model in a nude mouse with physiological saline (200 μL) or 200 μL of PEGylated WO_2.9_ NRs (20 mg/kg), respectively. After 1 h, the tumor in the mice was irradiated with a 980 nm laser at a power density of 0.35 W/cm^2^ for 10 min. The mice were euthanized after laser treatment. The tumors were removed, fixed in 4% paraformaldehyde at 4°C overnight, and embedded in paraffin for H&E staining and TUNEL staining.

The serial 5 μm thick sections were prepared and stained with haematoxylin/eosin (HE, Beyotime, China). Histology and morphology of tumor were observed under the Eclipse E800 microscope (Nikon, Japan). The DeadEnd Colorimetric TUNEL System from Promega (Mannheim, Germany) was used. Biotinylated nucleotides were incorporated at the 3′-OH DNA ends using the enzyme terminal deoxynucleotidyl transferase (TdT). Horseradish peroxidase-labeled streptavidin was then bound to these biotinylated nucleotides. Peroxidase activity was visualized using the liquid DAB substrate chromogen system (Dako, Hamburg, Germany). In case of antibody-TUNEL double staining, Cy2- labeled streptavidin (Dianova, Hamburg, Germany) was used. The number and percentage of TUNEL-positive cells were counted and determined by counting 1 × 10^3^ cells from five random selected fields.

### Photothermal ablation of HeLa cells *in vivo*

Twenty mice were divided to four groups randomly. The mice of the first group were intratumorally injected the saline solution (200 μL). The mice of the second group were intravenously injected with PEGylated WO_2.9_ NRs (20 mg·kg^−1^ body weight). The mice of the third group were only exposed to a 980 nm laser with a power density of 0.35 W/cm^2^ for 5 min every two days. The mice of the fourth group were intravenously injected with PEGylated WO_2.9_ NRs (20 mg·kg^−1^ body weight), then exposed to a 980 nm laser with a power density of 0.35 W/cm^2^ for 5 min every two days. The tumor size of all mice was measured and pictures of mice were taken every two days.

## Author Contributions

S.P.Y. and Z.G.Z. designed the experiments, B.K. and Z.G.Z. performed the experiments and data analysis, C.Y., M.W.W., W.L., Y.S., Y.J.Z. and H.Y. assisted with some of the experiments. S.P.Y., X.Y.S. and Z.G.Z. guided the work and wrote the paper.

## Supplementary Material

Supplementary Information Tungsten Oxide Nanorods: An Efficient Nanoplatform for Tumor CT Imaging and Photothermal Therapy
