# Supplementary material for: Tungsten Oxide Nanorods: An Efficient Nanoplatform for Tumor CT Imaging and Photothermal Therapy
Source: Sci Rep. 2014 Jan 13;4:3653. doi: 10.1038/srep03653 (PMC3888983; doi:10.1038/srep03653)
Supplement: Supplementary Information — Tungsten Oxide Nanorods: An Efficient Nanoplatform for Tumor CT Imaging and Photothermal Therapy [file srep03653-s1.doc]

Tungsten Oxide Nanorods: An Efficient Nanoplatform for Tumor CT Imaging and Photothermal Therapy

Zhiguo Zhou^1^, Bin Kong^1^, Chao Yu^1^, Xiangyang Shi^2^*, Mingwei Wang^3^, Wei Liu^1^, Yanan Sun^1^, Yingjian Zhang^3^, Hong Yang^1^, and Shiping Yang^1^*

^1^The Education Ministry Key Lab of Resource Chemistry and Shanghai Key Laboratory of Rare Earth Functional Materials, Shanghai Normal University, Shanghai 200234, Pepole’s Republic of China. ^2^College of Chemistry, Chemical Engineering and Biotechnology, Donghua University, Shanghai 210620, Pepole’s Republic of China. ^3^Department of Nuclear Medicine, Shanghai Cancer Center & Department of Oncology, Shanghai Medical College, Fudan University, Shanghai 200032, Pepole’s Republic of China. Correspondence and requests for materials should be addressed to Shiping Yang (email: shipingy@shnu.edu.cn) or to Xiangyang Shi (email: xshi@dhu.edu.cn)


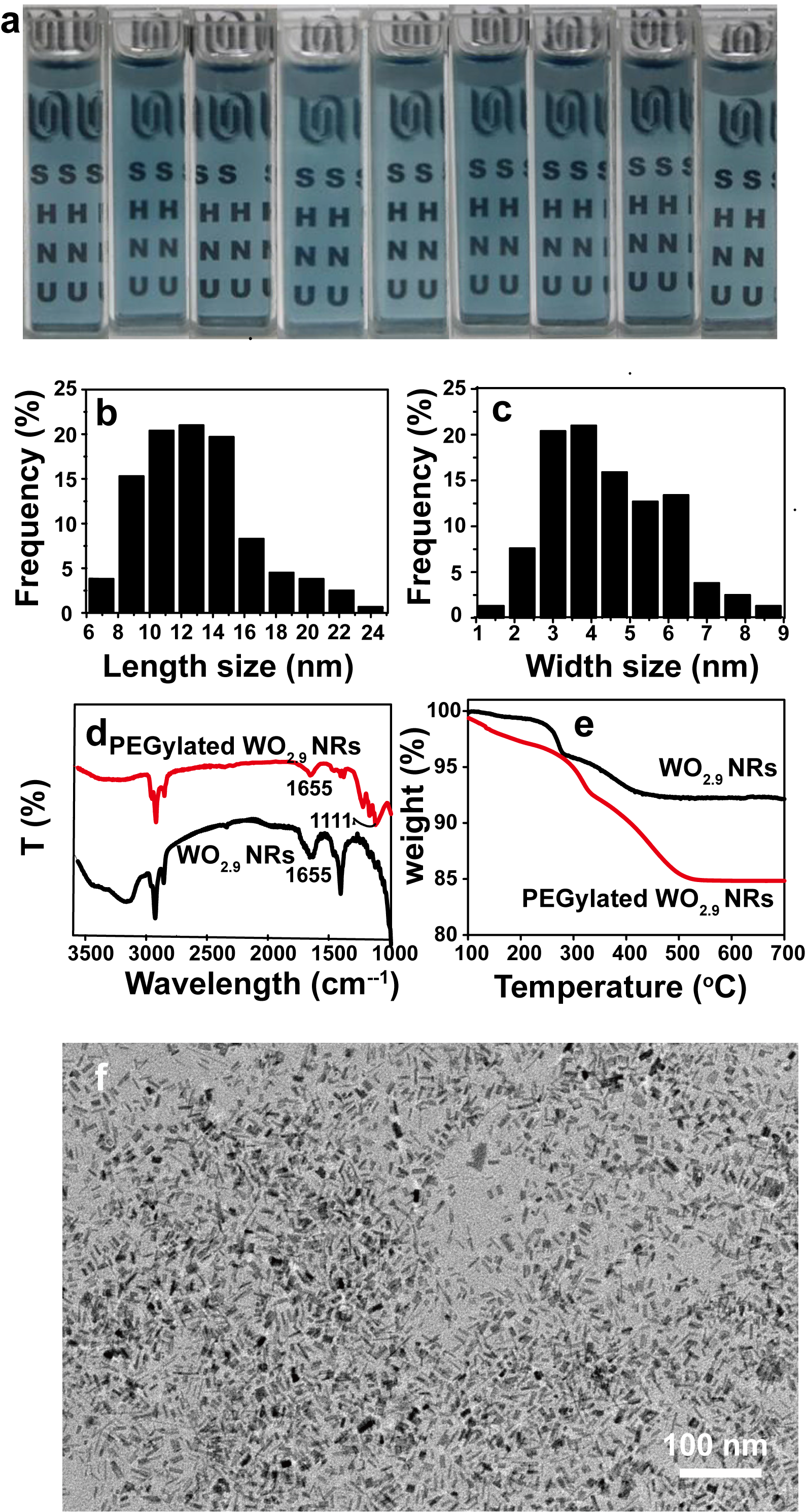


**Figure S1.** **Characterization of WO_2.9_ NRs and PEGylated WO_2.9_ NRs**. Photographs of WO_2.9_ NRs (100 μg/mL) in different solvents (a). From left to right: chloroform, toluene, dichloromethane, acetone, tetrahydrofuran, hexane, N, N-dimethyl formamide, dimethyl sulfoxide and cyclohexane. Length (b) and width (c) distribution of WO_2.9_ NR. FT-IR spectra of WO_2.9_ NRs and PEGylated WO_2.9_ NRs (d). TGA of WO_2.9_ NRs and PEGylated WO_2.9_ NRs (e). TEM image of PEGylated WO_2.9_ NRs (f).


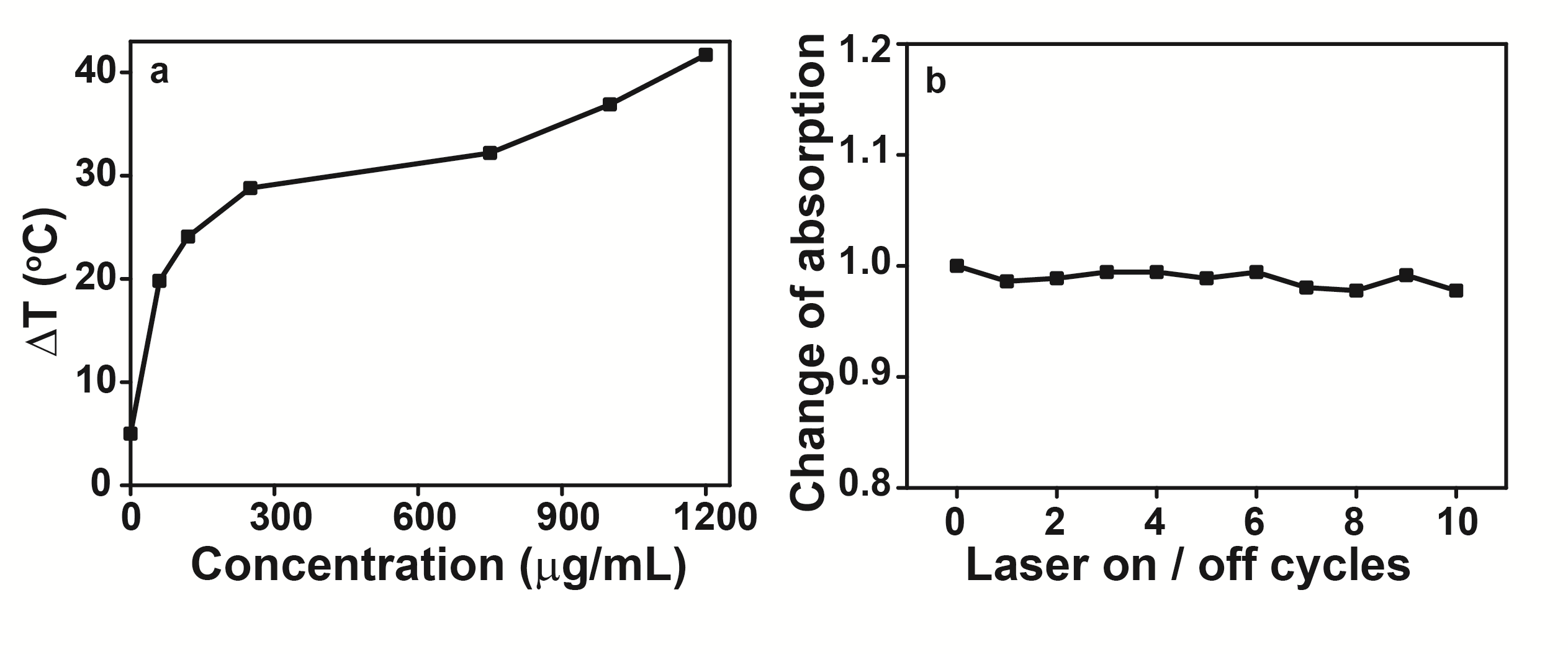


**Figure S2 Photothermal property of PEGylated WO_2.9_ NRs**. Plot of temperature change as a function of the concentration of PEGylated WO_2.9_ NRs under 980 nm laser irradiation (0.25 W/cm^2^) over a period of 10 min (a). Changes of absorbance at 980 nm after different Laser on/off cycles with a laser power density of 0.25 W/cm^2^ (b).


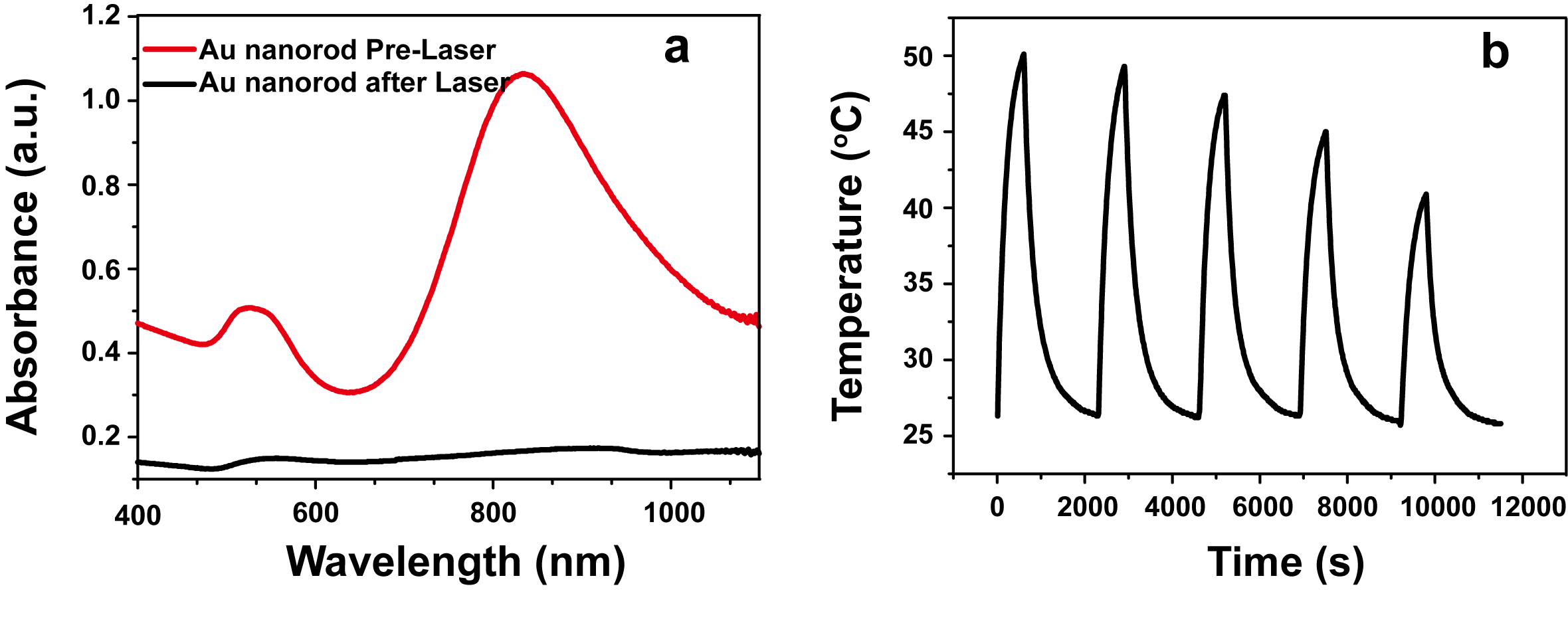


**Figure S3 Photothermal stability of CTAB-capped Au NRs.** (a) UV-Vis-NIR absorption spectra of CTAB-capped Au NRs in aqueous solution before (red) and after (black) the 980 nm laser irradiation over five LASER ON/OFF cycles (0.25 W/cm^2^). (b) Temperature change of the aqueous solution of CTAB-capped Au NRs over five LASER ON/OFF cycles (0.25 W/cm^2^).


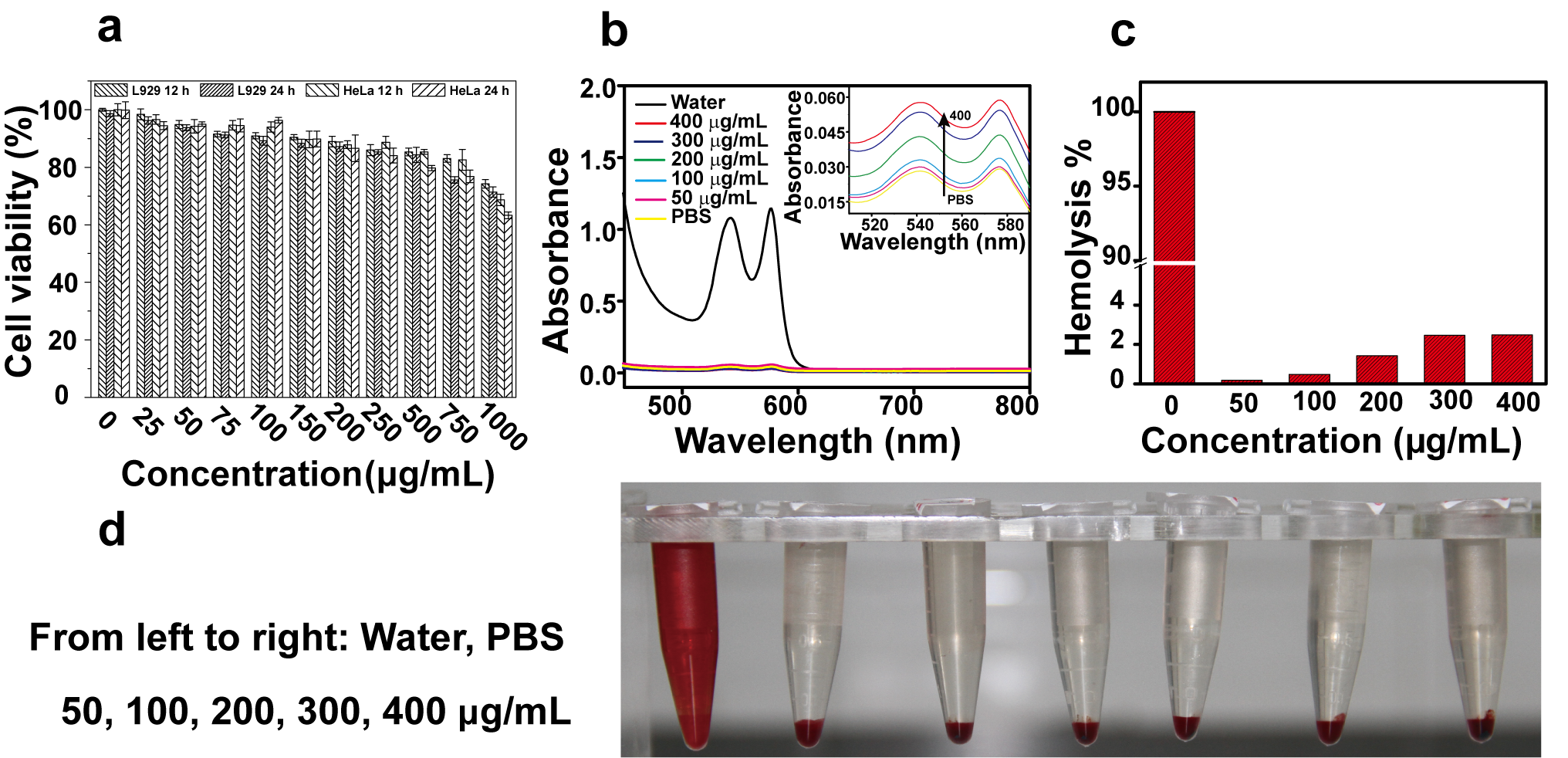


**Figure S4** **Biocompatibility of PEGylated WO_2.9_ NRs**. (a) MTT assay of viability of HeLa and L929 cells incubated with PEGylated WO_2.9_ NRs with different concentrations (0-1.0 mg/mL) for 12 and 24 h, respectively. (b) UV-Vis spectra of the HRBCs treated with PEGylated WO_2.9_ NRs with different concentrations (Water and PBS were used as positive and negative control, respectively). (c) Hemolysis percentage of PEGylated WO_2.9_ NRs with different concentrations. (d) Photo photographs of HRBC suspensions treated with water, PBS, and PEGylated WO_2.9_ NRs at different concentrations, followed by centrifugation .


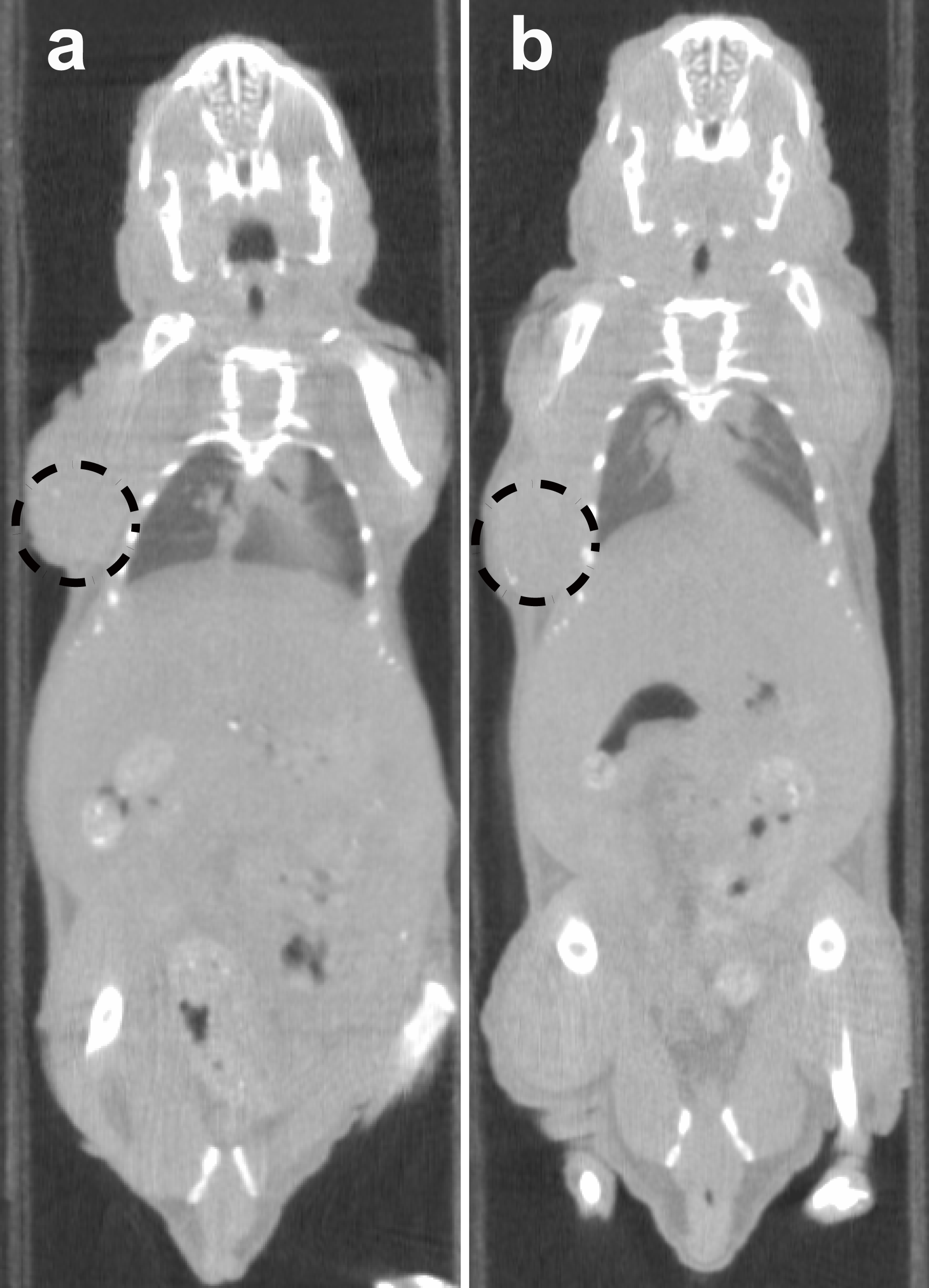


**Figure S5** CT images before (a) and after (b) intravenous injection with PEGylated WO_2.9_ NRs (20 mg/kg body weight).


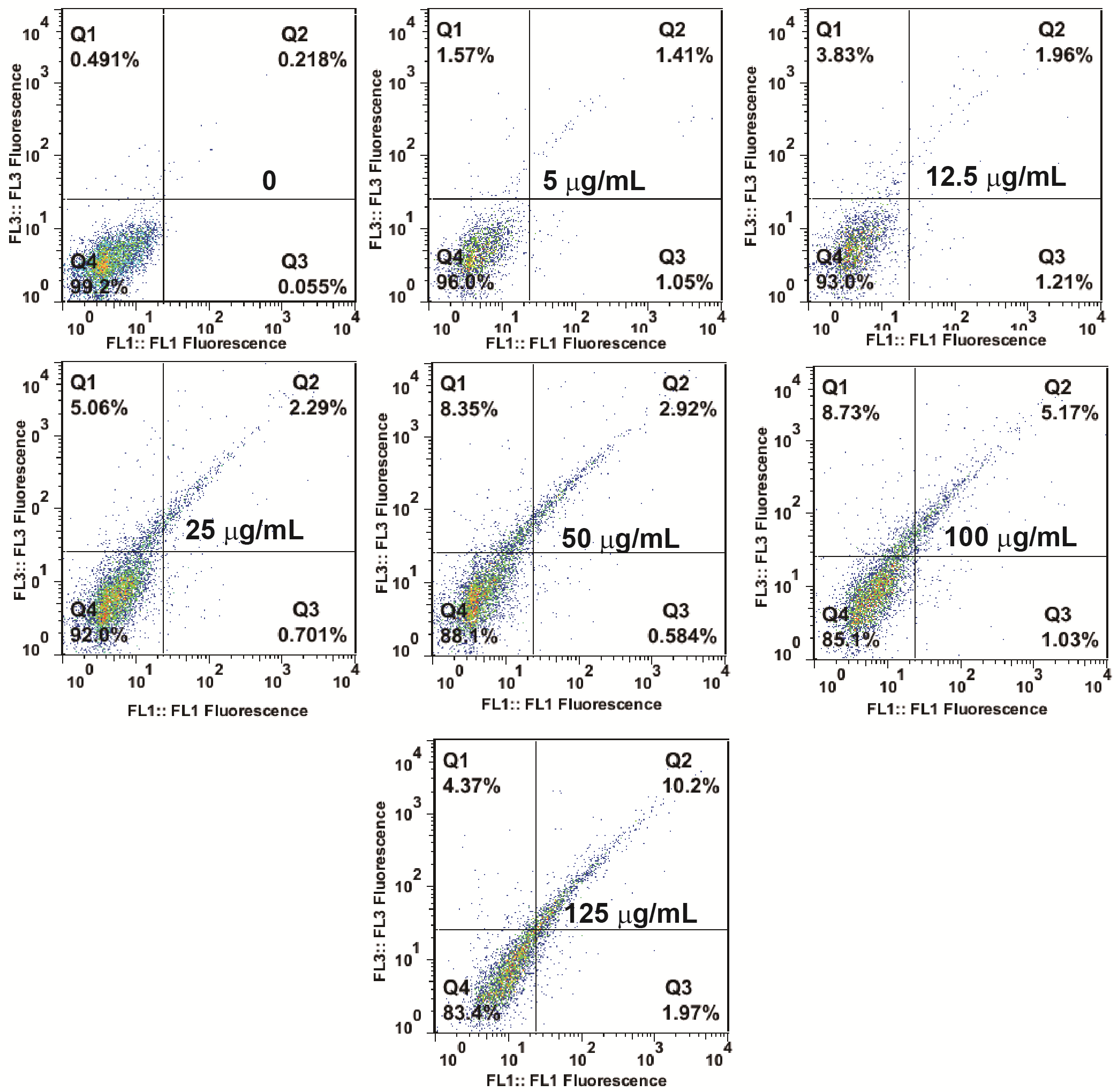


**Figure S6 The cell death mode using PEGylated WO_2.9_ NRs**. Apoptotic and necrotic cells for HeLa cells incubated with PEGylated WO_2.9_ NRs at different concentrations (0-125 μg/mL) detected by FACS.


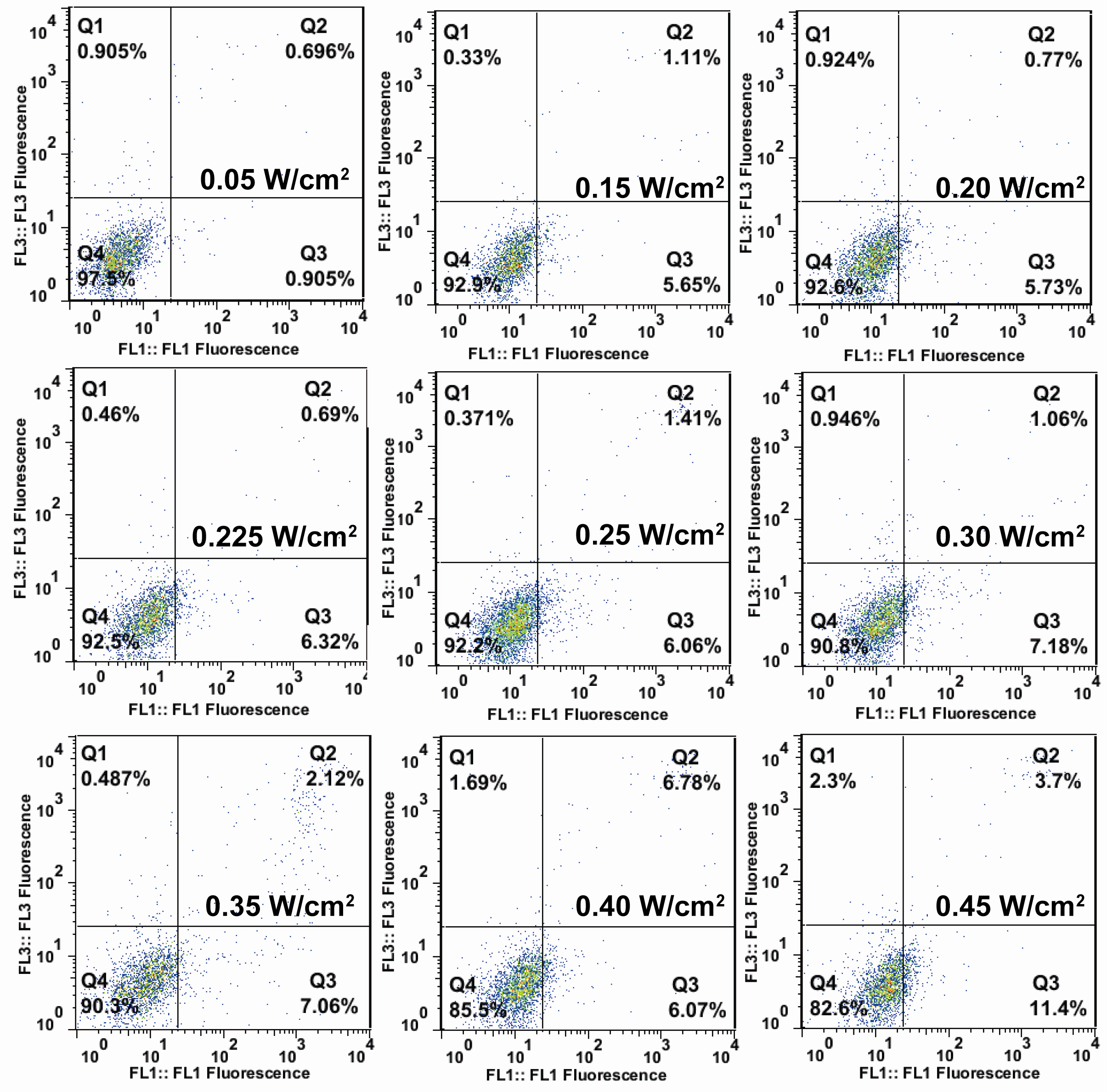


**Figure S7** **The cell death mode under 980 nm laser irradiation.** Apoptotic and necrotic cells irradiated by a 980 nm laser (0.05-0.45 W/cm^2^) detected by FACS.


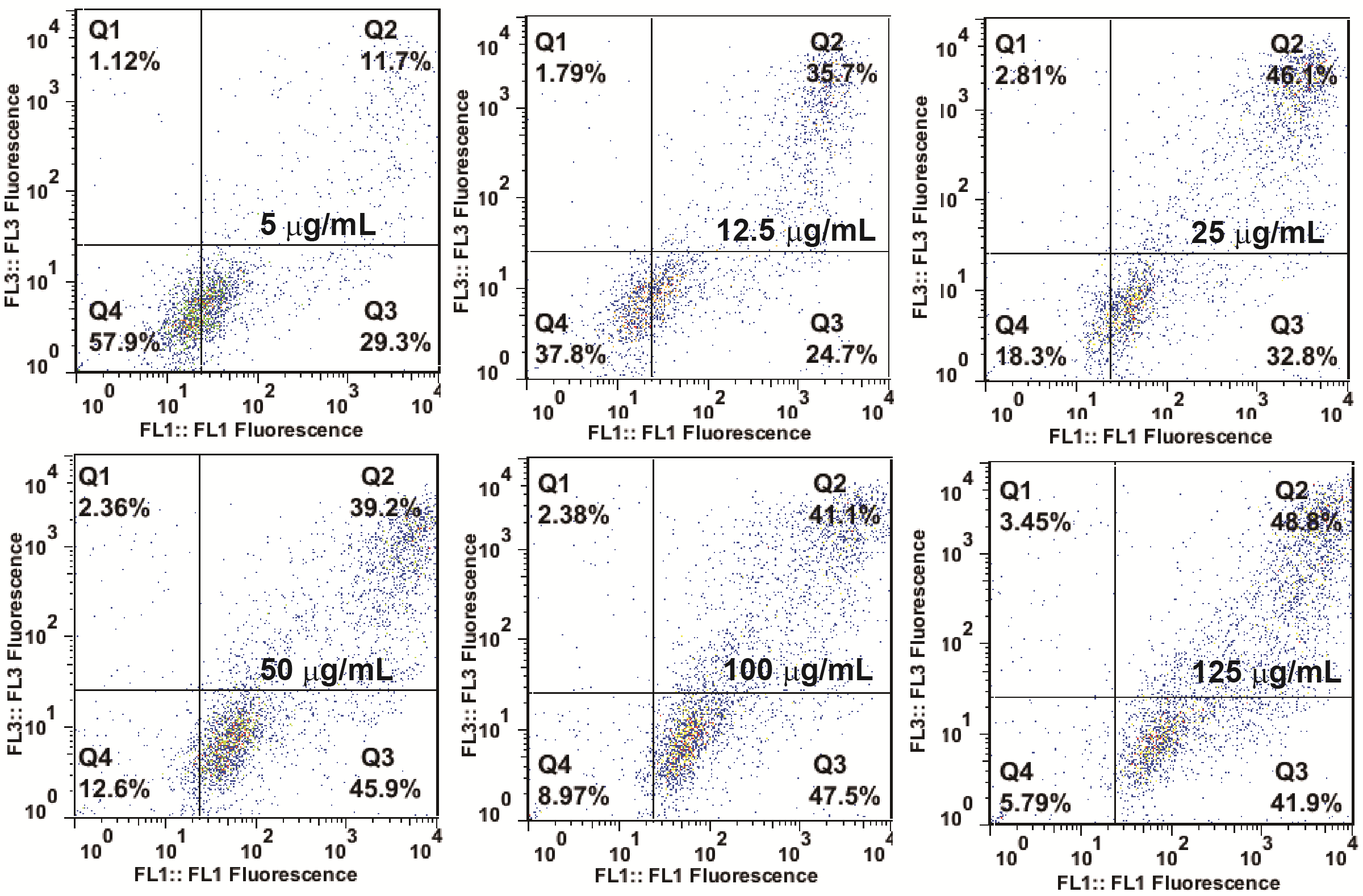


Figure S8 **The cell death mode using different concentration of PEGylated WO_2.9_ NRs under 980 nm laser irradiation**. Apoptotic and necrotic cells for HeLa cells incubated with PEGylated WO_2.9_ NRs (5-125 μg/mL) irradiated by a 980 nm laser (0.35 W/cm^2^) detected by FACS.


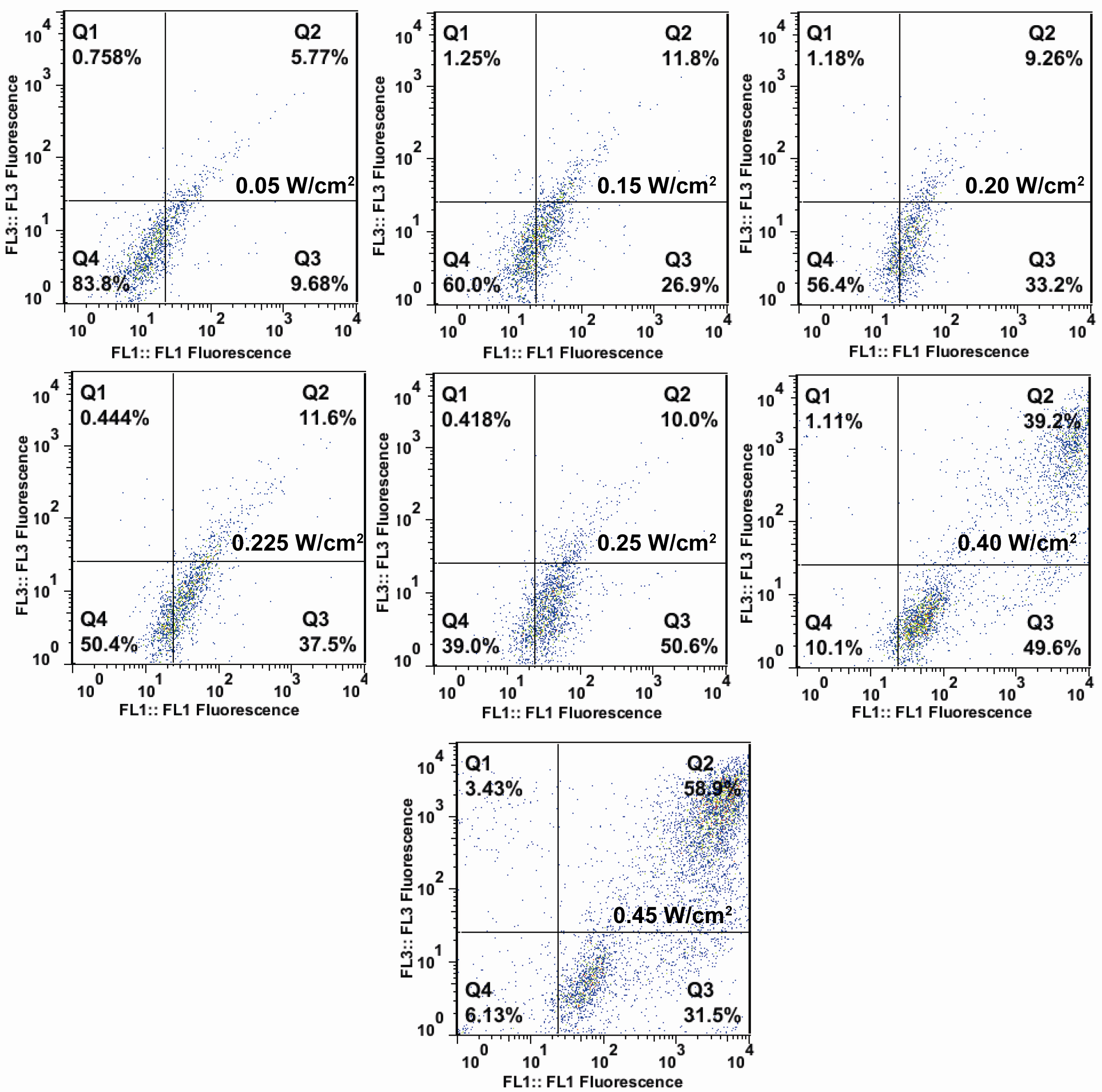


**Figure S9** **The cell death mode using PEGylated WO_2.9_ NRs with different power density of 980 nm laser irradiation**. Apoptotic and necrotic cells for HeLa cells incubated with PEGylated WO_2.9_ NRs (50 μg/mL) and irradiated by a 980 nm laser (0.05-0.45 W/cm^2^) detected by FACS.


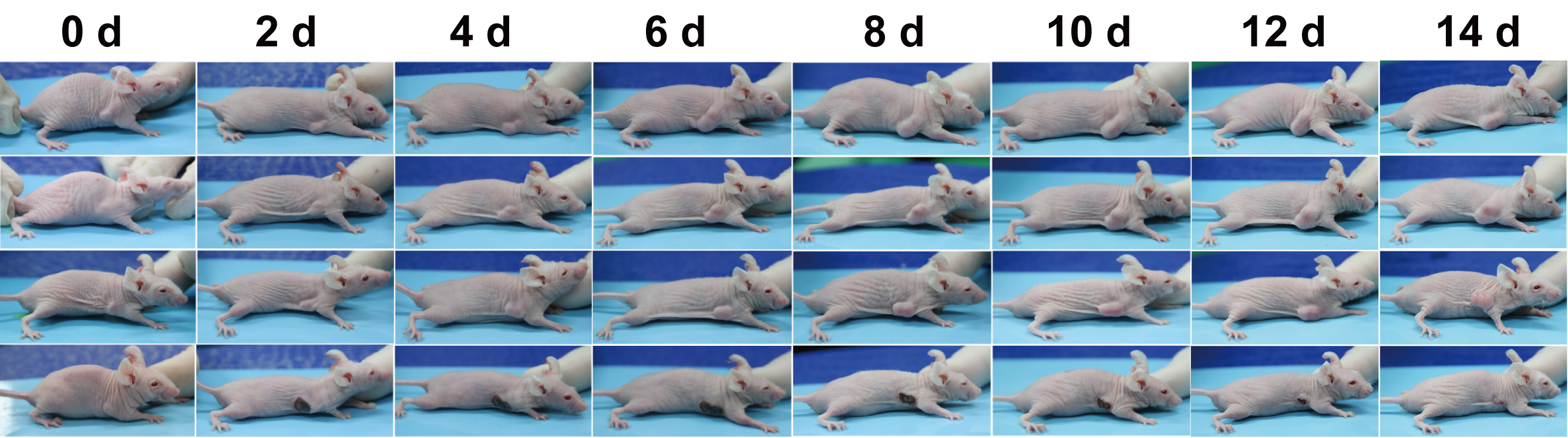


**Figure S10** Photographs of mice for all groups after photothermal therapy for 14 days. From top to bottom: mice after injection with the saline solution (200 µL), mice after injection with PEGylated WO_2.9_ NRs (20 mg/kg body weight), mice exposed to the 980 nm laser irradiation (0.35 W/cm^2^), mice after injection with PEGylated WO_2.9_ NRs (20 mg/kg body weight), then exposed to the 980 nm laser irradiation (0.35 W/cm^2^).
